# Mesenchymal Stem Cells Improve Medullary Inflammation and Fibrosis after Revascularization of Swine Atherosclerotic Renal Artery Stenosis

**DOI:** 10.1371/journal.pone.0067474

**Published:** 2013-07-03

**Authors:** Behzad Ebrahimi, Alfonso Eirin, Zilun Li, Xiang-Yang Zhu, Xin Zhang, Amir Lerman, Stephen C. Textor, Lilach O. Lerman

**Affiliations:** 1 Division of Nephrology and Hypertension, Mayo Clinic, Rochester, Minnesota, United States of America; 2 Division of Vascular Surgery, The First Affiliated Hospital of Sun Yat-sen University, Guangzhou, China; 3 Division of Cardiovascular Diseases, Mayo Clinic, Rochester, Minnesota, United States of America; University of Louisville, United States of America

## Abstract

Atherosclerotic renal artery stenosis (ARAS) raises blood pressure and can reduce kidney function. Revascularization of the stenotic renal artery alone does not restore renal medullary structure and function. This study tested the hypothesis that addition of mesenchymal stem cells (MSC) to percutaneous transluminal renal angioplasty (PTRA) can restore stenotic-kidney medullary tubular transport function and attenuate its remodeling. Twenty-seven swine were divided into three ARAS (high-cholesterol diet and renal artery stenosis) and a normal control group. Six weeks after ARAS induction, two groups were treated with PTRA alone or PTRA supplemented with adipose-tissue-derived MSC (10×10^6^ cells intra-renal). Multi-detector computed tomography and blood-oxygenation-level-dependent (BOLD) MRI studies were performed 4 weeks later to assess kidney hemodynamics and function, and tissue collected a few days later for histology and micro-CT imaging. PTRA effectively decreased blood pressure, yet medullary vascular density remained low. Addition of MSC improved medullary vascularization in ARAS+PTRA+MSC and increased angiogenic signaling, including protein expression of vascular endothelial growth-factor, its receptor (FLK-1), and hypoxia-inducible factor-1α. ARAS+PTRA+MSC also showed attenuated inflammation, although oxidative-stress remained elevated. BOLD-MRI indicated that MSC normalized oxygen-dependent tubular response to furosemide (-4.3±0.9, −0.1±0.4, −1.6±0.9 and −3.6±1.0 s^−1^ in Normal, ARAS, ARAS+PTRA and ARAS+PTRA+MSC, respectively, p<0.05), which correlated with a decrease in medullary tubular injury score (R^2^ = 0.33, p = 0.02). Therefore, adjunctive MSC delivery in addition to PTRA reduces inflammation, fibrogenesis and vascular remodeling, and restores oxygen-dependent tubular function in the stenotic-kidney medulla, although additional interventions might be required to reduce oxidative-stress. This study supports development of cell-based strategies for renal protection in ARAS.

## Introduction

Atherosclerotic renal artery stenosis (ARAS) remains among the most prevalent and important causes of secondary hypertension, and can accelerate renal injury towards fibrosis [1], [2]. Previous studies have shown that restoration of blood flow to the stenotic kidney by percutaneous transluminal renal angioplasty (PTRA) in patients with ARAS often fails to restore renal function [3], [4], [5] likely due to ongoing tissue injury, microvascular loss, and inflammation [4]. Therefore, adjunct therapies that decrease inflammation, promote angiogenesis, and attenuate tissue damage in the post-stenotic kidney hold the potential to enhance the outcome of PTRA in ARAS [6], [7]. However, renal injury is often evaluated in the entire kidney, which reflects mainly the renal cortex [7], [8], [9], whereas relatively little is known about the reversibility of medullary damage. We have recently shown that PTRA alone does not fully reverse medullary tissue damage in post-stenotic kidney [10]. We have further shown that intra-renal delivery of endothelial progenitor cells (EPC) combined with PTRA in a model of non-atherosclerotic RAS decreases medullary fibrosis and improves tubular transport function [10]. Nevertheless, even in that model with less damaged kidneys, EPC adjunct with PTRA did not restore medullary microvascular density or blood flow, possibly as a result of insufficient upregulation of angiogenic signaling.

Mesenchymal stem cells (MSC) are undifferentiated non-embryonic stem cells that can be isolated from a variety of tissues, and possess the ability to differentiate into a broad spectrum of cell lineages [11], [12]. In comparison to EPC, we have shown that MSC exhibit similar angiogenic properties, but greater anti-inflammatory properties [13]. Previous studies have shown in several kidney injury models that MSC promote tubular regeneration, and reduce inflammation and vascular injury [7], [11], [12], [14], [15]. In the cortex of ARAS pigs, we observed that MSC reduced oxidative stress, increased vascularity, and restored renal blood flow (RBF) and bioavailability of nitric oxide. Additionally, several studies have demonstrated that MSC up-regulated the expression level of anti-inflammatory cytokines such as interleukin (IL)-10 and attenuate renal fibrosis [12], [16]. These properties could potentially enhance their therapeutic efficacy compared to EPC. However, the ability of adjunct MSC, in addition to PTRA, to improve the function and the structure of the renal medulla has not yet been shown. Thus, this study was designed to test the hypothesis that in ARAS, adjunct MSC delivery during PTRA would improve medullary fibrosis, inflammation, tubular function, and microvascular density.

## Materials and Methods

### Ethics Statement

All animal procedures followed the Guide for the Care and Use of Laboratory Animals (National Research Council, National Academy Press, Washington, DC, 1996) and were approved by the Institutional Animal Care and Use Committee.

Twenty-seven female domestic pigs (48.3±1.5 kg) were studied for 16 weeks. Twenty of the animals were fed a high-cholesterol diet (to generate atherosclerosis) for the entire course of the study, starting six weeks before until ten weeks after induction of the stenosis. Unilateral ARAS was induced in these pigs by placing an irritant coil in one main renal artery [17], [18]. On the same day, a PhysioTel telemetry transducer (Data Science International, Arden Hills, MN, USA) was implanted in the femoral artery, and thereafter, mean arterial pressure (MAP) was continuously monitored and recorded. ARAS animals were randomized in three groups and six weeks later the degree of stenosis in each animal was determined using renal angiography. An untreated ARAS group (n = 7) underwent a sham procedure, and the other thirteen underwent PTRA and stenting under fluoroscopic guidance using a balloon-expandable stent [7], [19]. In addition, in the revascularized group, animals received after PTRA intra-renal infusion of either vehicle (n = 7) or adipose-tissue-derived MSC (n = 6, 10×10^6^ cells) over 5 min. Another group of pigs, fed with regular diet, underwent only sham procedures (angiography, saline infusion) and served as normal controls (n = 7).

Stenotic kidney function and oxygenation were determined four weeks later using multi-detector computed tomography (MDCT) and blood oxygen-level dependent (BOLD) magnetic resonance imaging (MRI), respectively. Prior to each in vivo study animals were anesthetized (Telazol 5 mg/kg and xylazine 2 mg/kg in saline), and anesthesia maintained with intravenous ketamine (0.2 mg/kg/min) and xylazine (0.03 mg/kg/min) (for CT), or inhaled 1–2% isoflurane (for MRI) throughout the course of imaging. BOLD images were collected before and 15 minutes after injection of furosemide (0.5 mg/kg) through an ear vein cannula. The BOLD index, R2*, at baseline and its change after furosemide, ΔR2*, were used as measures of tissue oxygenation and furosemide-suppressible oxygen-dependent tubular function, respectively [20].

Using contrast-enhanced MDCT, renal function was evaluated 3–4 days later in anesthetized pigs to calculate cortical and medullary volumes, regional renal perfusion, glomerular filtration rate (GFR) and RBF [21], [22].

Animals were euthanized with a lethal intravenous dose of sodium pentobarbital (100 mg/kg) a few days after the in vivo studies. Then the kidneys were removed and immersed in saline solution containing heparin. A lobe of tissue was perfused and prepared for micro-CT and another lobe was shock-frozen in liquid nitrogen and stored at −80°C or preserved in formalin for histology.

### Mesenchymal Stem Cells Preparation

Allogeneic MSC were collected and cultured from swine omentum fat (10 g), digested in collagenase-H for 45 min, filtered, and cultured in EGM-2 media for about 3 weeks [7]. The 3rd passage was collected and kept for up to 6 months in Gibco Cell Culture Freezing Medium (Life Technologies Co., Carlsbad, CA) at −80°C for transplantation. Cellular phenotype was examined in vitro with immuno-fluorescent staining of MSC positive for CD90, CD44, and CD105 and negative for CD14 and CD45, and cells were labeled with red fluorescent CM-DiI prior to injection, and 10^6^ cells/mL suspended in 10 mL of PBS. The immune-modulatory properties of MSC afforded the use of allogeneic cells with little concern about rejection [23].

### BOLD Imaging

BOLD imaging was performed on a 3T scanner (GE Medical Systems, Milwaukee, Wisconsin) using Fast Gradient Echo sequence with multiple echo times. Four to seven slices were collected axially in oblique planes using 16 echoes and MR parameters set to TR/TE/Flip Angle/FOV/Slice thickness/Matrix = 100 ms, 2.1–27 ms, 40°, 32 cm, 7 mm, 256×256. Entire acquisitions were performed during suspended respiration.

### MDCT Imaging

Contrast media injections during scanning were performed through a pigtail catheter, advanced through the left jugular vein to the superior vena cava. Following catheterization, the animals were moved to MDCT unit (Somatom Sensation 64; Siemens Medical Solutions, Forchheim, Germany). Initial tomographic positioning was determined with pilot scans. A bolus of iopamidol (0.5 ml/kg over 2 s) was then injected, and 140 consecutive scans acquired 3 seconds later, over approximately 3 minutes, as previously shown [7], [8], [9], [10].

Following the flow scan a volume study was performed after an additional contrast injection. Axial images were acquired at helical acquisition with thickness of 0.6 mm and resolution of 512×512, and reconstructed at 5 mm thickness. Kidney volumes were calculated from contrast-enhanced vascular phase images.

### Micro-CT for Microvascular Architecture

To prepare tissue for micro-CT imaging, the segmental renal artery perfusing a kidney lobe was cannulated ex-vivo and infused initially with heparin-saline (10 U/mL) and next by radio-opaque silicon polymer (Microfil, FlowTech, Carver, MA). Infusion was performed at the rate of 0.8 ml/min and under physiological pressure. Finally tissue was cut into small cubes, and scanning performed at 20 µm voxels-resolution.

### Data Analysis

All analyses were performed in Analyze™ (Biomedical Imaging Resource, Mayo Clinic, MN, USA) and MATLAB® (MathWork, Natick, MA, USA).

### MDCT

The volumes of the cortex and medulla were determined by estimating the total tomographic areas of each compartment and multiplying them by the thickness in 5 mm thick cross-sectional images. To calculate renal function and hemodynamics, the cortical and medullary signal attenuation vs. time curves were fitted to an extended Γ-variate model. Regional blood volumes and mean transit times were calculated to estimate cortical and medullary perfusion and blood flows (products of perfusion and the corresponding volumes). Total RBF was assessed as the sum of cortical and medullary flow. Finally, GFR was evaluated using the cortical curve and the slope of the proximal tubular curves, as previously shown [21].

### BOLD

Kidneys were sampled using entire kidney region of interest. Pixel-by-pixel R2* maps were generated by fitting the logarithms of the decaying MR signal against corresponding echo times and calculating the slope. The histogram of the population of estimated R2* values was plotted against R2* scale, and the regions representing the cortical and medullary compartments separated, as recently shown [24]. An average medullary R2* was calculated, and the difference between the baseline and post-furosemide R2* values, ΔR2*, was used as a quantitative measure of oxygen-dependent tubular transport.

### Micro-CT

Using Analyze™, micro-CT images were presented as 3-dimensional volumes of the microvasculature. Medullary microvascular density (vascular volume fraction) was calculated by dividing the contrast-agent filled vasa recta volume, calculated from micro-CT images, by the volume of the entire region of outer medulla containing these microvessels [10], using 100–200, 20 µm-thick cross-sectional slices.

### MSC Localization

MSC, labeled with CM-DiI prior to the injection, were tracked in frozen 5 µm thick cross-sectional slices under fluorescence microscopy. To determine the location of engraftments, DAPI (nuclear stain) and cytokeratin (tubular marker) co-stains were used. MSC were counted both in the cortex and medulla in low power fields. The engraftment ratio in the medulla to the cortex was calculated, as shown before [7].

### Kidney Tissue Studies

Frozen medullary tissue was isolated, and standard Western blotting protocols were performed by pulverizing and homogenizing it in chilled protein extraction buffer. The lysate was used for immunoblotting against swine specific antibodies for vascular endothelial growth factor (VEGF) (dilution 1∶200), VEGF receptor (FLK-1) (dilution 1∶200), hypoxia-inducible factor (HIF)-1α (dilution 1∶500), NAD(P)H-oxidase p47 (dilution 1∶200), matrix metalloproteinase (MMP)-2 (dilution 1∶500), IL-10 (dilution 1∶500), and tumor necrosis factor (TNF)-α (dilution 1∶200), to investigate mechanisms involved in renal neovascularization, tubular oxygen consumption, fibrogenesis and inflammation. GAPDH served as a loading control, except for MMP-2 expression, which was quantified by normalizing the active to pro-MMP.

Renal vascularization, fibrosis and inflammation mediators were also quantified in 5 µm slides stained for H&E, trichrome, CD68, CD163+ macrophages, Arginase (Arg)-1 (a marker of trophic M2 macrophages) and monocyte chemotactic protein (MCP)-1, and oxidative stress in 30 µm dihydroethidium (DHE) stained slides. Medullary capillaries, tubules and neutrophils were counted at 100× magnification in H&E stained slides using an ApoTome microscope (Carl ZEISS SMT, Oberkochen, Germany). Capillaries were identified by the presence of lumen, red blood cells and/or an endothelial cell lining, and the ratio of capillary number to tubules was calculated [25]. In addition, microvascular density was assessed by immunoreactivity of von Willebrand factor (vWF) in colorimetric stained slides. Tubular injury was scored on a 1–5 scale (1: <10%, 2∶10–25%, 3∶26–50%, 4∶51–75% and 5: >75% injury) using 40× H&E slides based on tubular dilation, atrophy, cast formation, sloughing tubular endothelial cells or thickening basement membrane [26].

### Statistical Analysis

Results are presented in mean±SEM format. Paired Student *t-*test was used for comparisons within groups and ANOVA for comparison among groups followed by unpaired t-test. For *p* values ≤0.05 differences were considered significant.

## Results

Six weeks after stenosis induction, the degree of stenosis in the ARAS, ARAS+PTRA and ARAS+PTRA+MSC groups was comparable (77±7%, 78±5% and 75±4%, respectively) and MAP was similarly elevated (ANOVA p = 0.42) (Table 1). Four weeks after PTRA (residual stenosis <10%) MAP declined in both ARAS+PTRA and ARAS+PTRA+MSC (p = 0.0006 and p = 0.006 vs. ARAS, respectively). Serum creatinine in ARAS+PTRA remained elevated similar to ARAS, but was not different from normal in ARAS+PTRA+MSC. Renal volume, RBF and GFR decreased in ARAS compared to normal. In ARAS+PTRA and ARAS+PTRA+MSC, medullary volumes were restored to normal values, but medullary blood flow remained lower than normal in both groups, although it tended to be higher in ARAS+PTRA+MSC compared to ARAS+PTRA alone (p = 0.07). PTRA alone did not improve GFR, but PTRA+MSC restored GFR to a value not different from normal.

**Table 1:** Single kidney function (mean±SEM) in pigs with atherosclerotic renal artery stenosis ARAS after 10 weeks of stenosis, 4 weeks after intervention with percutaneous transluminal renal angioplasty (PTRA), with or without adjunct Mesenchymal Stem Cells (MSC).

Parameters	Normal	ARAS	ARAS+PTRA	ARAS+PTRA+MSC
Body Weight (kg)	51.6±2.1	51.3±4.1	47.3±3.3	50.0±3.9
Serum Creatinine (mg/dl)	1.32±0.05	1.98±0.01*	1.84±0.1*	1.41±0.04^#$^
Total Cholesterol (mg/dl)	92.2±17.9	488.8±81.7*	412.0±133.7*	395.7±97.0*
Mean Arterial Pressure (mmHg)				
-6-Weeks	94.1±6.6	139.4±4.2*	136.7±4.2*	131.5±5.1*
-10-Weeks	97.3±5.3	131.5±5.3*	102.7±2.6#	103.2±7.0#
Volume (mL)				
-Medulla	21.7±1.3	18.5±0.8*	19.0±2.0	21.3±2.8
-Cortex	111.6±4.8	75.2±4.0*	85.4±4.6*	104.8±6.7^#$^
Blood Flow (mL/min)				
-Medulla	83.3±10.3	48.6±8.6*	32.2±13.8*	58.7±6.8*
-Cortex	525.4±24.6	331.3±41.9*	382.9±40.9*	478.6±53.2#
Renal Blood Flow (mL/min)	608.7±29.5	379.9±49.5*	415.1±44.7*	537.3±59.0#
Glomerular Filtration Rate (mL/min)	83.6±9.1	50.8±6.8*	50.7±4.3*	68.2±5.3^#$^

* p≤0.05 vs. Normal,

# P≤0.05 vs. ARAS.

$ p≤0.05 vs. ARAS+PTRA,

### Medullary Tubular Oxygen-dependent Function

**Figure 1:**
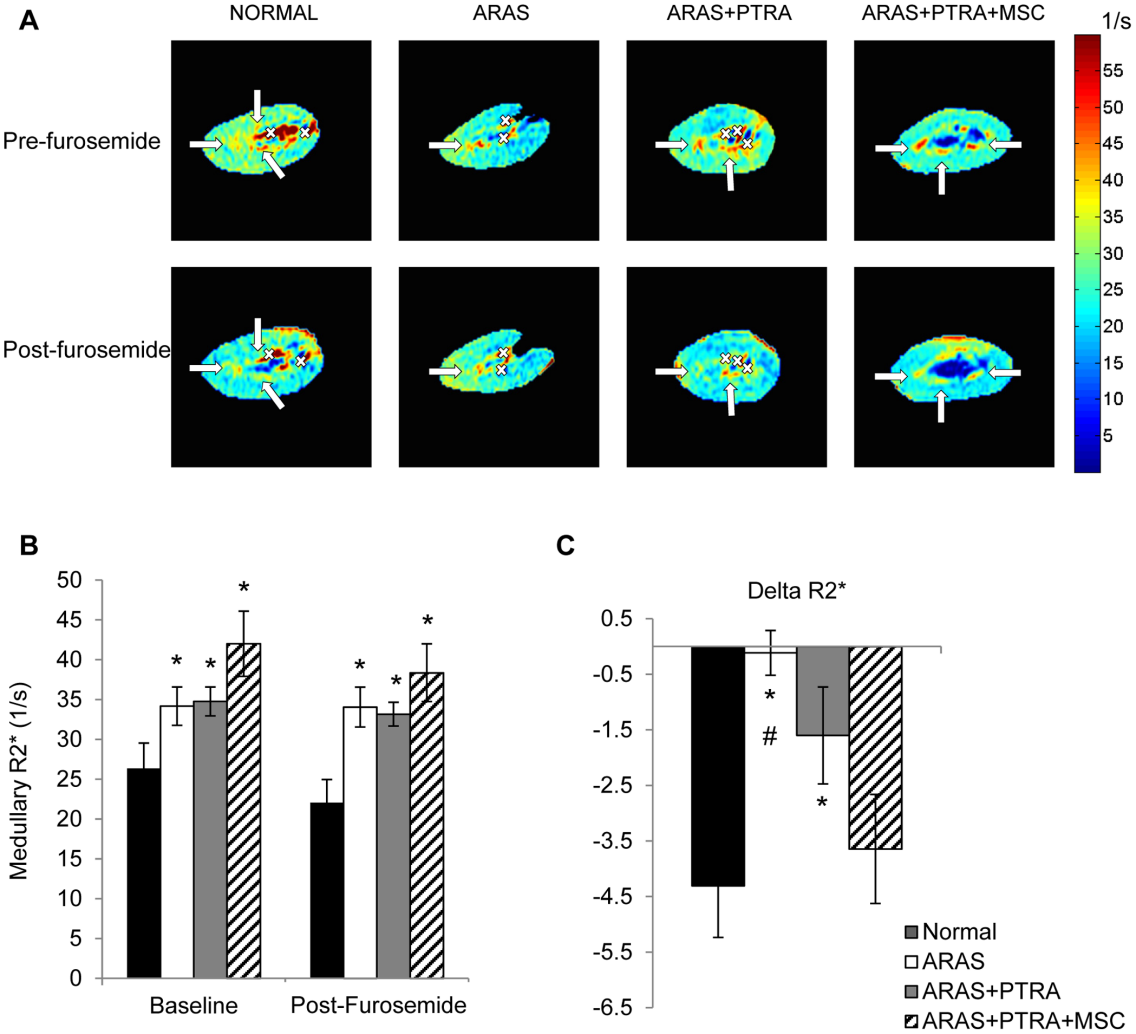
BOLD parametric map in Normal, ARAS, ARAS+PTRA and ARAS+PTRA+MSC pigs. Arrows indicate the medullary regions. Vessels localized from T2*-images have been crossed-out to differentiate from hypoxic regions (A). Medullary R2* was higher both at baseline and after furosemide in all ARAS groups compared to normal (B). The response to furosemide remained blunted in ARAS, and partial response was detected in ARAS+PTRA, but tubular oxygen-dependent function was restored in ARAS+PTRA+MSC (C) (*p≤0.05 vs. Normal, ^#^p≤0.05 ARAS+PTRA+MSC).

Basal medullary R2* values were elevated in all ARAS groups compared to normal and tended to be higher in ARAS+PTRA+MSC compared to the other two ARAS groups (Figure 1, p = 0.07 vs. ARAS and p = 0.08 vs. ARAS+PTRA). After furosemide, the medulla remained more hypoxic than normal in all ARAS animals, and R2* was not statistically different among them (Figure 1A–B). The change in R2*, as a measure of oxygen-dependent tubular transport function, indicated significantly attenuated response in ARAS and ARAS+PTRA compared to Normal. However, the degree of response in ARAS+PTRA+MSC was significantly enhanced compared to ARAS, tended to be higher than ARAS+PTRA (p = 0.08), and was not different from Normal (Figure 1C).

### Medullary Morphology

**Figure 2:**
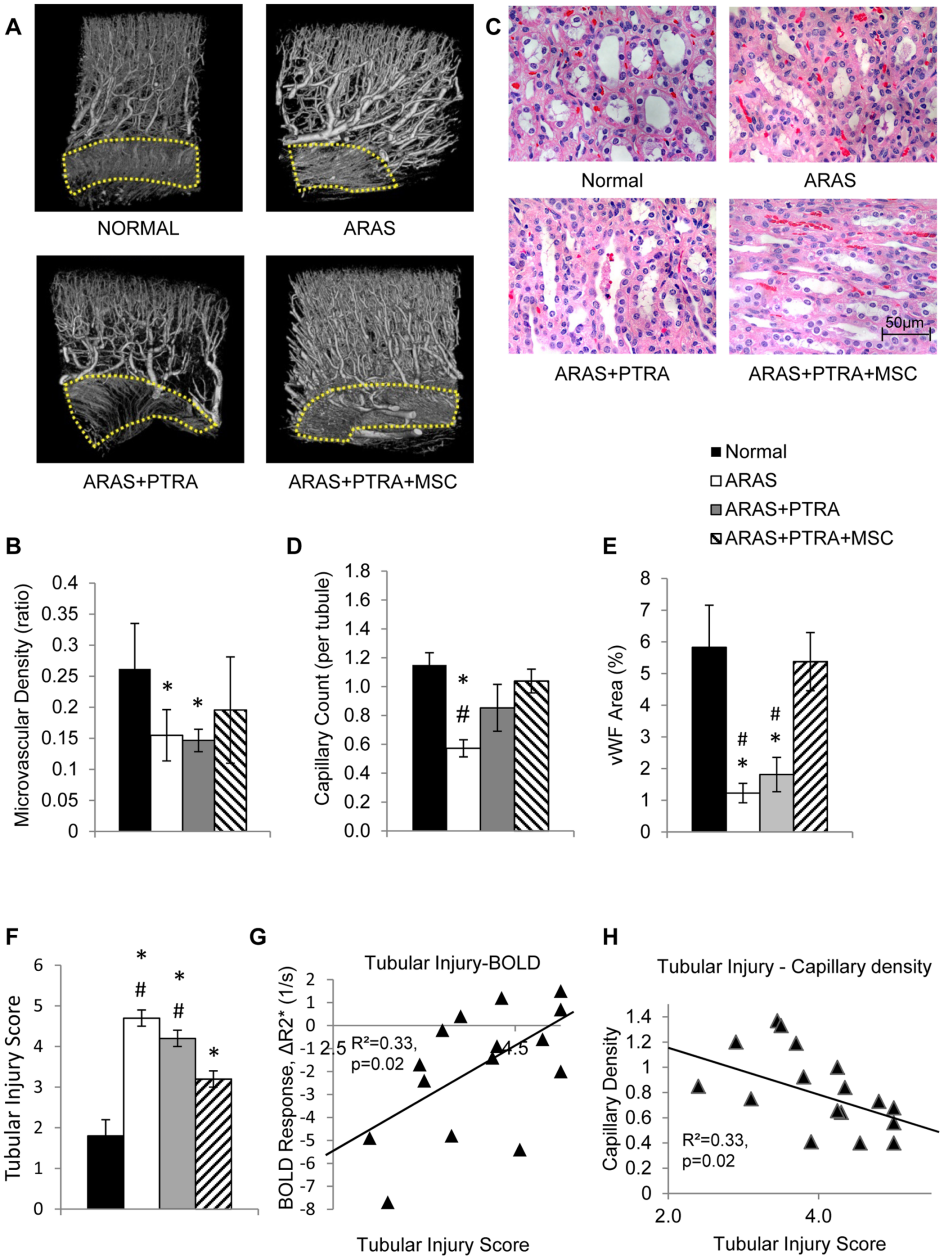
Medullary neovascularization and tubular injury. Medullary regions outlined in dashed-line were used to calculate vasa recta density from micro-CT (A). The medullary microcirculation in stenotic kidneys. Vasa recta density was calculated from micro-CT as the fraction of tissue volume (B). Representative H&E-stained medullary tubules (x40 images) (C). The number of capillaries per tubule was calculated from H&E stained slides at x100 (D), and vWF quantified from vWF stained slides (x20) (E), Semi-quantitative tubular injury score (1–5 scale) calculated from corresponding H&E images (F) Medullary BOLD response to furosemide (G) and capillary density (H) showed moderate but significant correlation with tubular injury (*p≤0.05 vs. Normal, ^#^p≤0.05 ARAS+PTRA+MSC).

Micro-CT revealed a decrease in vasa recta density in ARAS and ARAS+PTRA, but not in ARAS+PTRA+MSC compared to normal (Figure 2A, 2B). Capillary count also showed significantly lower densities in ARAS (p<0.001 vs. normal), which tended to remain reduced in ARAS+PTRA (p = 0.08), but was similar to Normal in ARAS+PTRA+MSC (Figure 2.D). Moreover, medullary vWF expression, a marker of endothelial cells density, was restored to normal in ARAS+PTRA+MSC, but remained diminished in ARAS and ARAS+PTRA. Semi-quantitative medullary tubular score showed significantly greater injury in all ARAS animals compared to normal but higher scores in ARAS and ARAS+PTRA than ARAS+PTRA+MSC (both p<0.05) (Figure 2F). Finally, medullary BOLD response to furosemide did not correlate with capillary density but both showed moderate but significant correlations with tubular injury score (Figure 1G–H).

### Tissue Studies

**Figure 3:**
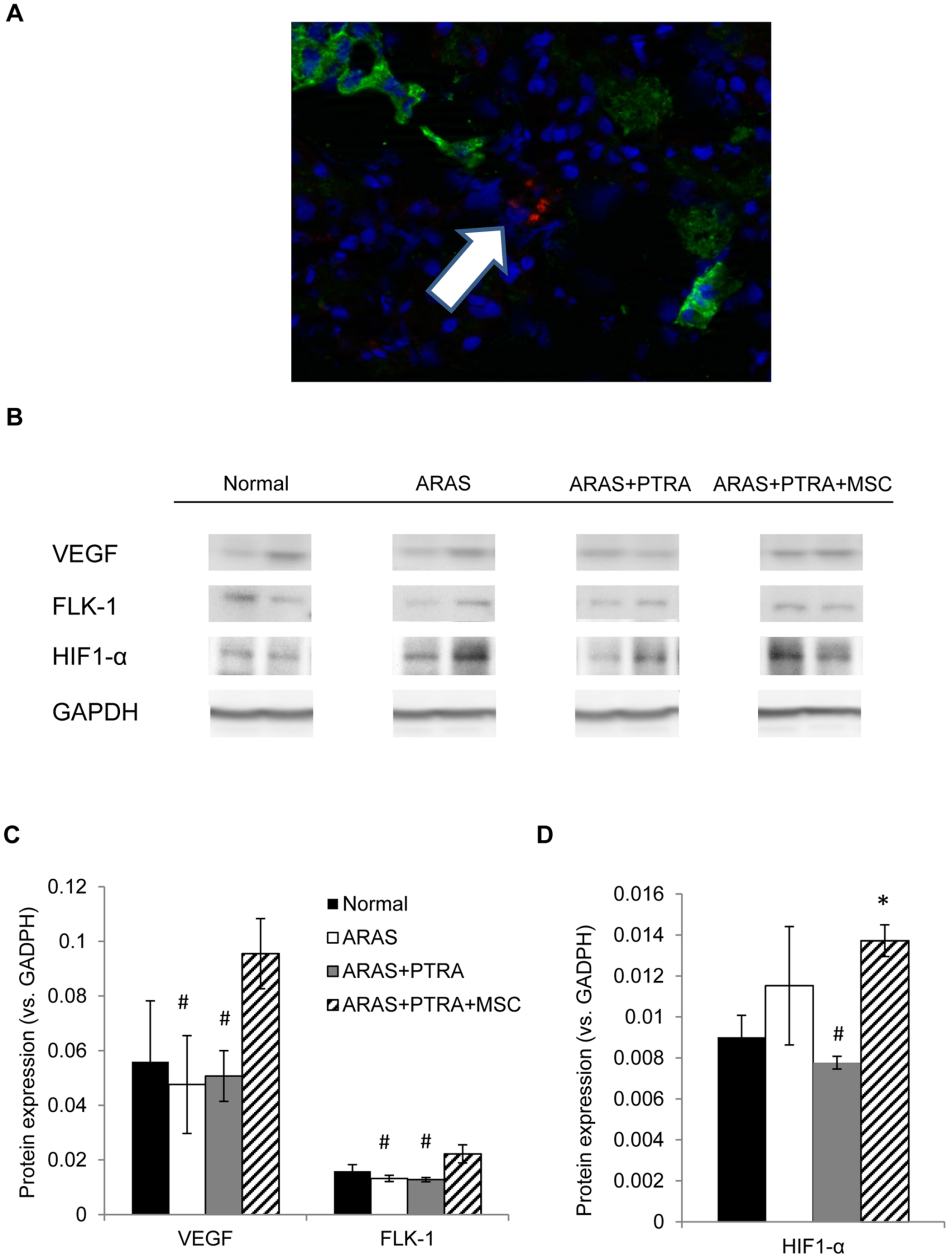
Angiogenic factors and cell engraftment. MSC, labeled with CM-DiI (red), were localized in medullary slides co-stained with DAPI nuclear stain (blue) and the tubular marker cytokeratin (green). MSC were observed mainly engrafted in the medullary interstitium (A). Representative immunoblots (B) and quantification of medullary expression of the angiogenic factors VEGF, FLK-1 (C) and hypoxia inducible factor, HIF1-α (D). (*p≤0.05 vs. Normal, ^#^p≤0.05 vs. ARAS+PTRA+MSC).

**Figure 4:**
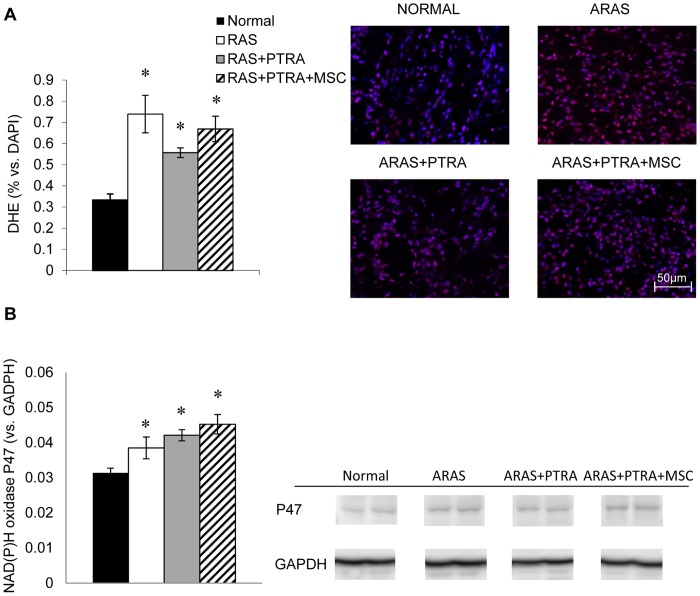
Markers of oxidative stress in the medulla. Co-staining (x40) of DHE (red) and the nuclear marker DAPI (blue) was used to quantify their ratios (A) in Normal, ARAS, ARAS+PTRA and ARAS+PTRA+MSC. Medullary expression of NAD(P)H oxidase P47 was elevated in all ARAS medullas (B) (*p≤0.05 vs. Normal).

In the stenotic kidney medulla of ARAS+PTRA+MSC, 4 weeks after administration MSC were detected engrafted mainly in the interstitium (Figure 3A), and their average ratio to MSC in the cortex was approximately 1∶5 [7]. Medullary expression of VEGF and its receptor FLK-1 was increased in ARAS+PTRA+MSC compared to ARAS and ARAS+PTRA, and tended to be higher than normal (p = 0.07, and p = 0.08, respectively). Despite increased angiogenic signaling in ARAS+PTRA+MSC, medullary expression of HIF1-α, an index of hypoxia, was upregulated in this group, but not in ARAS+PTRA, compared to Normal (Figure 3B,D). Medullary NAD(P)H oxidase p47 expression and DHE staining were similarly elevated in all ARAS groups (Figure 4).

**Figure 5:**
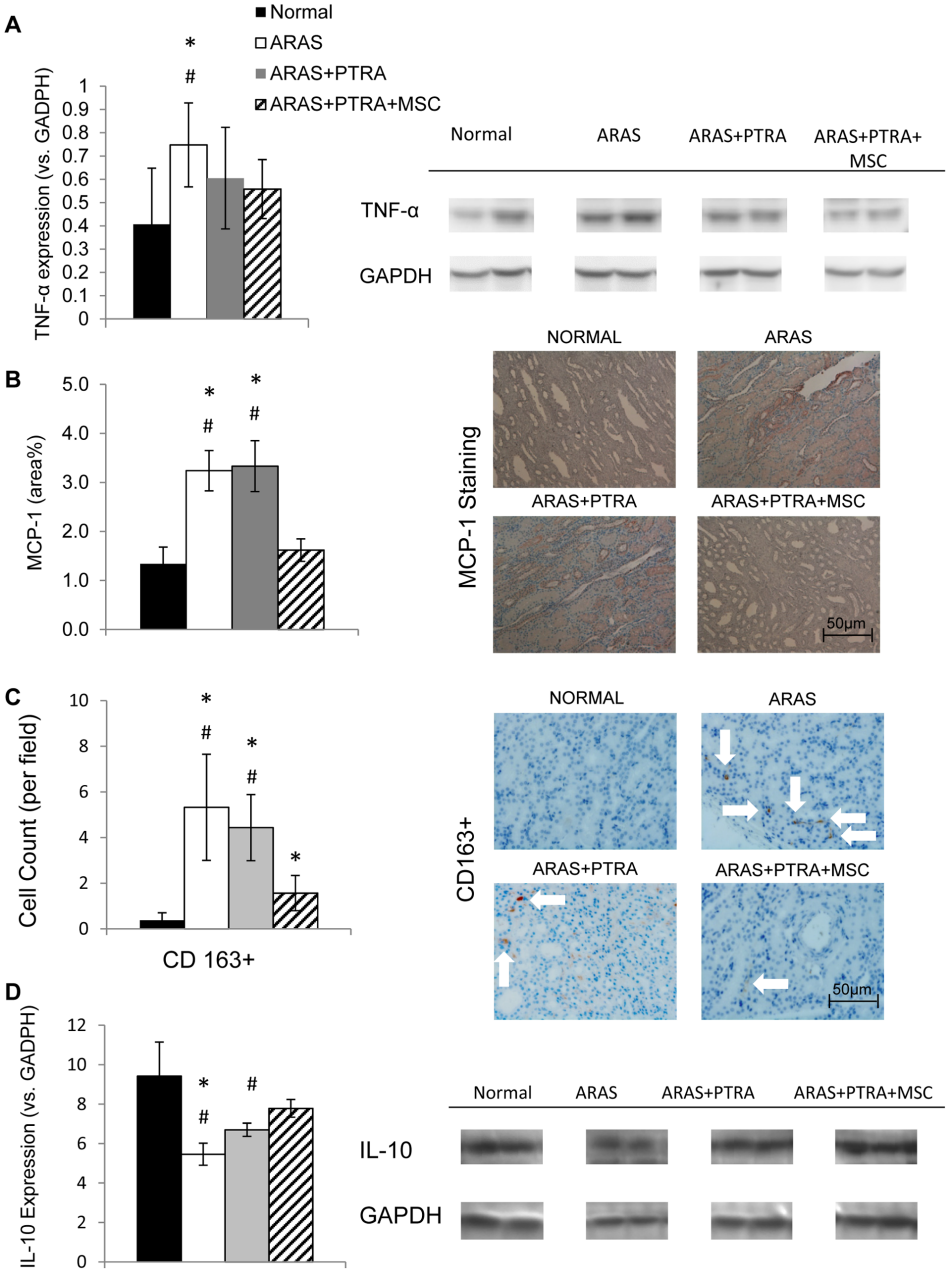
Medullary markers of inflammation. TNF-α (A) and MCP-1 (B) expression, and CD163+ macrophage count (C). The anti-inflammatory factor IL-10 showed a significantly increased expression in ARAS+PTRA+MSC vs. the two other ARAS groups (D) (*p≤0.05 vs. Normal, ^#^p≤0.05 ARAS+PTRA+MSC).

Expression of the inflammatory mediator TNF-α was significantly elevated in ARAS compared to Normal and to ARAS+PTRA+MSC. Additionally, MCP-1 immunoreactivity was elevated in ARAS and ARAS+PTRA, but did not differ between ARAS+PTRA+MSC and normal animals. Although medullary counts of CD163+ macrophages remained elevated in ARAS+PTRA+MSC compared to Normal, they were lower than in ARAS and ARAS+PTRA. Furthermore, the expression of the anti-inflammatory cytokine IL-10 in ARAS+PTRA+MSC was augmented vs. the other two ARAS groups (Figure 5).

**Figure 6:**
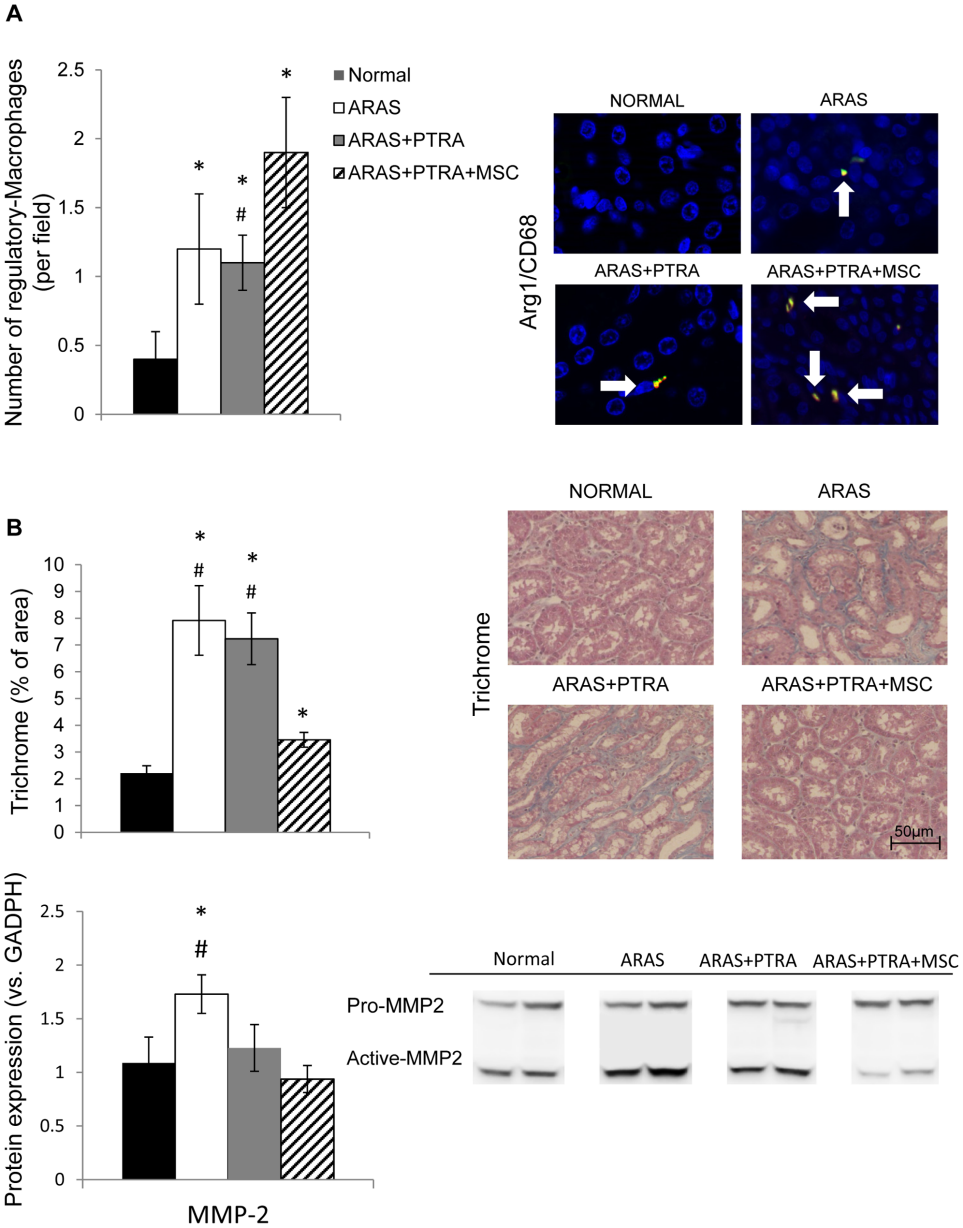
Quantification of regulatory M2 macrophages in CD68/Arg-1 double-stained slides. The number of M2 macrophages in ARAS+PTRA+MSC was significantly higher than Normal and in ARAS+PTRA groups (A). Medullary fibrosis. Trichrome staining and MMP-2 expression decreased in ARAS+PTRA+MSC compared to ARAS (B) (*P≤0.05 vs. Normal, ^#^p≤0.05 ARAS+PTRA+MSC).

The CD68^+^/Arg1^+^ co-staining showed that the population of trophic-macrophages was significantly higher in both ARAS and ARAS+PTRA vs. Normal (Figure 6) and further higher in ARAS+PTRA+MSC compared to ARAS+PTRA. In all RAS groups, results revealed very limited number of neutrophils in the microvasculature and nearly none infiltrated in the tissue.

Medullary tubulointerstitial fibrosis (trichrome-staining) was greater than Normal in all experimental groups, but was significantly blunted in ARAS+PTRA+MSC compared to ARAS and ARAS+PTRA. Similarly, MMP-2 was only elevated in ARAS, but was attenuated compared to ARAS in ARAS+PTRA+MSC but not in ARAS+PTRA (Figure 6).

## Discussion

The current study showed that PTRA with adjunct MSC delivery restores renal medullary vascularization and attenuates tubular injury and inflammation substantially more effectively than PTRA alone. Restoration of main vessel blood flow by PTRA was associated with partial recovery of medullary volume and reduction in capillary loss, but medullary tubular responses to furosemide remained blunted, and GFR and medullary blood flow remained as low as in untreated ARAS animals. By contrast, ARAS+PTRA+MSC treated kidneys were characterized by enhanced expression of angiogenic markers, reduced tubulointerstitial fibrosis, and reduced inflammatory markers. These changes occurred despite higher oxidative stress (reflected by DHE and p47) and more evident medullary hypoxia (defined by elevated medullary R2*). Addition of MSC to PTRA enhanced the response of medullary oxygenation after furosemide, consistent with increased tubular solute transport.

Although obstruction of the main renal artery and reduction in RBF for any reason can produce renal parenchymal damage, the atherosclerotic milieu in ARAS adds an independent adverse impact on the progression of the renal injury and its hallmarks [17], [18], such as endothelial dysfunction, fibrosis and hypoxia. These observations suggest that deleterious factors beyond the stenosis associated with the atherogenic process play important roles in compromising renal function and potential recovery in ARAS. Medullary volume and flow declined in ARAS in this study to greater extent than previously observed in RAS, possibly related to microvascular vasoconstriction and rarefaction [2].

A decrease in blood and consequently oxygen supply observed in ARAS is often associated with inflammation, tubulointerstitial fibrosis and microvascular rarefaction. Our micro-CT and capillary count suggested loss of vasa recta and their network of peritubular capillaries with no change in VEGF expression. Medullary deoxyhemoglobin as reflected by BOLD measurements rose, associated with a rise in markers of oxidative stress (DHE and p47) in all ARAS groups. Remarkably, tissue hypoxia as reflected by HIF-1α expression was not measurably increased in ARAS compared to normal.

PTRA alone had limited success in decelerating the parenchymal changes of ARAS, as fibrosis remained higher than normal and similar to ARAS, and BOLD imaging indicated only partial response to furosemide. Considering the similar GFR in ARAS and ARAS+PTRA, this response was less likely related to solute delivery than a partial improvement in tubular function, possibly as a result of a small increase in capillary density after PTRA. The direct correlation between tubular function (BOLD response) and injury score is consistent with this notion. Although medullary blood flow remained impaired, increased peritubular capillary density identified by histology suggested that PTRA reduced damage to the peritubular capillaries. Nevertheless, microvascular and capillary densities tended to remain low, and baseline R2* values indicated significantly lower tissue oxygenation in this group. Notably, the insignificant correlation between capillary density and BOLD response may suggest that medullary tubular function is regulated by factors other than blood supply alone (e.g. GFR). Additionally, ARAS+PTRA manifested tendency for an increase anti-inflammatory (IL-10) and decrease pro-inflammatory (TNF-α) mediators, which might have blunted tubular injury compared to ARAS. Despite limited tissue oxygenation and increased oxidative stress, HIF-1α expression remained unchanged, perhaps due to the blunted tubular transport function in ARAS+PTRA compared to ARAS. Moreover, although medullary blood flow was similar in ARAS and ARAS+PTRA, due to their higher peritubular capillary density the tubules in ARAS+PTRA might be spatially closer to capillaries and receive higher oxygen supplies.

In contrast to PTRA alone, adjunct MSC improved medullary vascularization and tubular function and reduced inflammation and tubulointerstitial fibrosis. The vWF staining, microvascular density, and peritubular capillary counts strongly suggest that the microvasculature in the medulla of ARAS+PTRA+MSC was relatively protected or restored, possibly secondary to up-regulated expression of growth factors like VEGF and its receptor, FLK-1. Nevertheless, the basal R2* and HIF1-α expression in this group indicated higher level of tissue hypoxia compared to the other two ARAS groups. We interpret this as likely secondary to the greater oxygen demand imposed by improved GFR and solute transport, in the presence of yet limited medullary perfusion and increased oxidative stress. Reduced medullary blood flow despite preserved microvasculature has been previously attributed to a regulatory mechanism involving reactive oxygen species in the medullary thick ascending limb (mTAL), tubular-vascular crosstalk [27], [28]. Conversely, increased tubular transport function despite residual tubular injury may account for the increased oxidative stress in ARAS+PTRA+MSC group, as in the presence of concentrated tubular solutes, activation of transport in mTAL during hypoxia may increase intracellular concentrations of superoxide [29] as a result of engagement of anaerobic glycolysis instead of aerobic transport function. In ARAS and ARAS+PTRA, on the other hand, the increase in oxidative stress might be linked to sustained inflammation. In this study, several inflammatory markers were lower in ARAS+PTRA+MSC, particularly CD163+ macrophage infiltration, perhaps due to prominent anti-inflammatory characteristics of MSC (e.g. increase in IL-10 expression) as well as reduced expression of the chemoattractant MCP-1. Additionally, we observed that MSC increased the presence of M2 phenotype macrophages, which are often linked to tissue repair. This may account for the lesser tubulointerstitial injury and reduced fibrosis compared to ARAS and ARAS+PTRA. Furthermore, lower vascular and interstitial remodeling in the MSC group was associated with diminished expression of MMP-2, which reflects restoration of matrix turnover and therefore reduced scar tissue. These anti-inflammatory properties may also underlie the greater efficacy of MSC in preserving the medullary microcirculation in the stenotic kidney compared to EPC.

The mechanism of tissue regeneration after administration of MSC has been the focus of great interest [30], [31], [32]. Some studies suggest that repair of injured tissues rarely involves cell replacement and MSC differentiation, whereas the paracrine action of MSC often accounts for amelioration of injury. While paracrine signaling may be mediated by release of cytokines and growth factors into the extracellular environment, recent studies have implicated MSC shedding of membrane microvesicles, which provide channels for transport and communication among the cells, and allow exchange of proteins, lipids, and RNA [33]. Such microvesicles may contain growth factors like VEGF, which is necessary for generation and maintenance of the renal microcirculation, or anti-inflammatory cytokines like IL-10, benefits conferred by MSC that involve delivery of trophic substances to the renal tissue.

Limitations: Due to the limitations of experimental studies on large animals our group sizes were limited. In our model ARAS develops over a period of 16 weeks, which is shorter than the usual duration of the disease in humans, and might influence its interaction with pathological factors involved in disease progression. Nevertheless, the chain of events and mechanisms of tissue injury in our ARAS model closely resemble that in humans. In addition, the spatial resolution of the micro-CT imaging technique that we used to calculate medullary microvascular density is close to the limit needed to evaluate individual vasa recta. Furosemide may increase renal vascular resistance in some models, but has no significant effect on RBF in swine unilateral RAS [20], arguing against a hemodynamic influence on tissue oxygenation and assessment of tubular function. Special care was also taken to avoid partial volume error, yet we cannot completely rule out the possibility of some errors. The optimal timing and dose of MSC that yield the greatest renoprotective benefit will need to be determined in future studies.

In conclusion, PTRA alone elicited only partial recovery of renal medullary tubular transport function, microvascular density and inflammation in ARAS. Adjunct delivery of MSC+PTRA improved GFR as well as medullary vascular densities and oxygen-dependent tubular transport. Delivery of MSC increased medullary R2* levels, enhanced VEGF expression and reduced MCP-1 compared with PTRA alone. These changes were associated with reduced CD163+ cells in the post-stenotic kidney, increased IL-10 and M2 macrophages, and lower interstitial fibrosis. Adjunctive PTRA+MSC improved medullary blood flows and recovery of kidney function, possibly as a result of microvascular repair and anti-inflammatory effects of MSC. This study therefore extends the value of MSC as adjunctive therapy to restore medullary structure and function in ARAS after PTRA.
